# Bayesian Hierarchical Modeling for Variance Estimation in Biopharmaceutical Processes

**DOI:** 10.3390/bioengineering12020193

**Published:** 2025-02-17

**Authors:** Sonja Schach, Tobias Eilert, Beate Presser, Marco Kunzelmann

**Affiliations:** 1 CMC Statistics Development Biologicals, Boehringer Ingelheim Pharma GmbH & Co. KG, Birkendorfer Straße 65, 88397 Biberach an der Riß, Germany; beate.presser@boehringer-ingelheim.com (B.P.); marco.kunzelmann@boehringer-ingelheim.com (M.K.); 2 CMC Statistics BioPharma, Boehringer Ingelheim Pharma GmbH & Co. KG, Birkendorfer Straße 65, 88397 Biberach an der Riß, Germany; tobias.eilert@boehringer-ingelheim.com

**Keywords:** heteroskedastic process modeling, random-effect variances, meta-analysis, biopharmaceutical process variability, Bayesian borrowing, location-scale model

## Abstract

Determining process variances in biopharmaceutical manufacturing is challenging due to limited data availability. To address this, we introduce a Bayesian hierarchical model designed for meta-analysis of process variance. This approach can improve process variance estimation by integrating data from multiple products, providing more reliable estimates of critical quality attributes in cases of data scarcity. Additionally, our model aids in evaluating process models, ensuring quality in process development. The paper demonstrates the new method using a simulation study, showcasing its potential to leverage historical data for both upstream and downstream phases of future CMC drug development. The new statistical model has great potential to expedite the market introduction of therapies while ensuring patient safety, allowing new treatments to reach patients more quickly without compromising quality or efficacy.

## 1. Introduction

In the development of new biopharmaceutical drugs, the urgency to accelerate the process for earlier patient access is accompanied by the need to save on large-scale batch runs during the developmental phase. While these savings are crucial in reducing the time and high costs required for batch production, they lead to data scarcity, posing a significant challenge for CMC (Chemistry, Manufacturing, and Controls) bioprocess development. A critical aspect of biopharmaceutical development is establishing comprehensive process knowledge based on the determination of the variability of critical quality attributes (CQAs). Understanding and accounting for process variability at defined input-parameter set points is fundamental. It ensures regulatory compliance, product quality, process robustness, cost efficiency, and effective risk management. Providing accurate process variability estimates is essential for statistical analysis during the developmental phase, ultimately determining the specifications of future products.

However, traditional statistical methods for estimating process variability often fall short when data are limited [1,2], leading to biased or inaccurate variance estimates. Currently, no alternative statistical approach exists that enables the meta-analysis of hierarchical process data as a predictive model for new products while accounting for product variance as a random effect. This study primarily focuses on addressing this issue by demonstrating a novel methodological approach designed to handle small sample sizes in bioprocess development. It provides a description of the hierarchical model, which is designed to be applicable to a multilevel data structure arising from batch and cycle (i.e., measures per batch) dependencies.

Data scarcity often presents significant challenges, highlighting the need for innovative statistical methods to enhance reliability [3]. This issue is also prevalent in clinical statistics. Borrowing information from historical data is a currently discussed topic that is particularly beneficial in scenarios with limited patient numbers [4]; in the context of early-phase clinical trials; or in rare diseases where patient numbers are limited and, thus, only small sample sizes are available [5]. This scarcity can lead to unreliable estimates and hinder the ability to draw meaningful conclusions. Empirical Bayesian borrowing methods address this issue by incorporating information from observed data into the analysis as a prior, allowing for more robust statistical inferences. The concept of Meta-Analytic-Predictive (MAP) priors [6,7,8] is a key tool in this context. MAP prior approaches can inform a current study by leveraging data from multiple historical studies. MAP priors combine data to create a prior distribution that reflects accumulated knowledge. This prior distribution represents the initial belief about a parameter based on meta-analytic data (e.g., from previous products) before observing any current data. This prior belief is then updated with new data (e.g., from a new product), supporting the analysis as an informative prior. In this way, Bayesian borrowing techniques can lead to more accurate statistical inferences [7]. They can improve the reliability of clinical trial outcomes, especially when historical and current data are similar, such as clinical control group data, leading to better-informed decisions. Furthermore, an empirical Bayesian prior approach (e.g., power prior approach [9]) allows for a more flexible and data-driven way to determine how much weight should be given to historical information from previous projects, ensuring that the prior information is neither overemphasized nor underutilized.

Particularly common in the context of clinical drug development, meta-analysis methodologies allow for the combination of results from multiple clinical trials to draw more robust conclusions. A critical assumption in meta-analysis is the homogeneity of effect sizes, which underpins statistical testing for between-study heterogeneity. This testing assesses whether the included studies are sufficiently similar to be combined. Various methodological approaches exist to estimate the variance between studies. Variability among studies is often captured by random-effect modeling, incorporating a random-effect variance component ($\tau^{2}$), assuming that all studies are sampled from the same population. Estimates of heterogeneity variance and its confidence interval can be provided through several methods, including the DerSimonian and Laird method or variants such as the Paule–Mandel method and approaches by Hartung and Makambi and Sidik and Jonkman. Langan et al. [10,11] and Veroniki et al. [12] provided a comprehensive review of these methods. However, such methods perform poor on a small number of studies [13]. Bayesian approaches, as suggested by Bodnar et al. [14], offer a promising alternative, providing more accurate interval estimates.

In a meta-analysis setting, when pooling data with clustered samples, such as those from the same groups—for instance, studies and treatment groups within the context of clinical trials analog to products and batches within the context of CMC manufacturing—it is crucial to account for these dependencies. Ignoring such dependencies can lead to flawed inferences due to the underestimation of standard errors, as noted in [1,2]. To address this, a nested variance model can be employed, allowing for the estimation of variance components at each level within the hierarchy [15]. This approach effectively manages heteroscedasticity and ensures more accurate results. Such a nested multilevel model design considers variances both within groups and between groups as random effects, thereby partitioning the total variance and offering insights into the contribution of each hierarchical level to the overall variability. Nested random-effect models are powerful tools for understanding hierarchical data structures. However, they do not provide variance estimates across groups, which limits their use as predictive models. Assuming that all groups have the same variance (homoscedasticity), the model is essentially averaging the variances, which can obscure the true variability when violating this assumption [16]. The hierarchical nature of these models can lead to either wider or narrower confidence intervals for variances due to the additional levels of uncertainty, i.e., less precise estimates of the variance components at multiple levels (within-group and between-group variability).

Location-scale models [17,18,19,20] offer a robust framework for understanding and modeling data variability. In the context of meta-analysis, location-scale models are particularly valuable, as they account for variability both within and between groups. These models are especially useful when data exhibit differences in both location (mean) and scale (variance) across groups. The location parameter represents the central tendency, while the scale parameter captures dispersion or variability. By estimating these parameters, location-scale models effectively manage heterogeneity, providing a probability distribution for the variance of a response variable. This approach is well-suited to the hierarchical nature of meta-analytic data, where individual studies (or batches in bioprocess development) may have different mean values and variability.

This integration of location-scale modeling assumptions within a new multilevel meta-analysis model is seen to have great potential in the development and manufacturing of biopharmaceuticals to enhance the ability to draw meaningful conclusions from small sample sizes. It allows for the pooling of information across processes being developed on a comparable platform. A model that appropriately considers the data hierarchy can thereby improve the robustness of statistical inferences to process variance estimated based on limited data. In this paper, we present a Bayesian hierarchical model that extends the principles of location-scale modeling to accommodate multilevel data structures and allows for the prediction of population variance distribution. The model is able to describe heterogeneity of variances across products as random effects considering both between-batch variance (similar to between-study variance in meta-analysis) and within-batch variance. In this way, the total product variance, as the sum of both variance components, is described as originating from a lognormal distribution.

This has significant relevance for biopharmaceutical process development.

Currently, there is no other robust statistical method that can fully address these challenges of small sample sizes, making this an ongoing area of investigation. By incorporating both location and scale parameters, our model can better capture the inherent variability in biopharmaceuticalplatform processes, leading to more reliable and precise estimates and providing a more accurate representation of the data. The general model structure is introduced, facilitating Bayesian inference on model parameters. In the presented simulation study, we systematically examined the impact of dataset sample sizes and ratios of variance components. By doing so, we validated the model and demonstrated the robustness and accuracy of model predictions. By leveraging Bayesian hierarchical modeling, this approach offers the novel advantage of allowing for meta-analytic study of variance of CQA measures. The resulting model prediction is proposed to serve as a meta-analytic predictive prior for new products, informing early phases of development where only a few large-scale runs are available. In this way, the method aims to enhance the efficiency of bioprocess development, enabling the faster delivery of new therapies to patients while ensuring quality and efficacy. Thus, it provides a promising solution to the industry’s pressing need for cost-effective and timely data analysis.

## 2. Materials and Methods

### 2.1. Model Implementation

#### 2.1.1. Hierarchical Data Structure

The proposed model for variance meta-analysis is designed specifically for application to process data in biopharmaceutical development.

**Figure 1:**
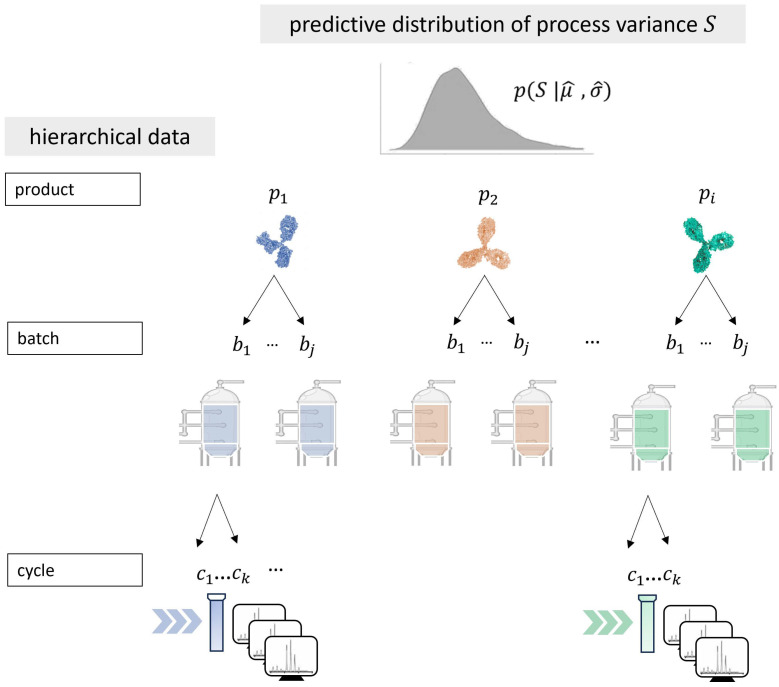
Hierarchical structure of process data and predictive random-variance model. A biopharmaceutical platform process in CMC development describes similar products ($p_{i}$), considered as a population level in the meta-analysis. Individual products are produced in batches, with multiple cycle measurements for CQAs. The resulting data structure appropriately combines within-batch and between-batch variance within the statistical model. The predictive distribution ($p(S|\hat{\mu },\hat{\sigma })$) is used for cross-product variance estimates of the platform process.

The hierarchical data structure of CQA measures of a platform process is illustrated in Figure 1. The upstream manufacturing of various molecules (e.g., antibodies) is carried out in fermenters. Subsequently, the downstream process involves purifying the cell culture from the fermenter as batch material. Typically, the measurements of individual CQAs for different products (*i*) come from various batches (*j*), which can include several cycles of measurements (*k*), representing an additional hierarchical level. The hierarchical nature of the data has to be appropriately considered within a product meta-analysis. The predictive model of process variance based on historical platform data results from the Bayesian posterior of model parameters (see Section 2.1.2). These parameters define the predictive distribution of process variance (*S*), which quantifies platform process variance (*S*) for future products.

#### 2.1.2. Model Implementation

**Figure 2:**
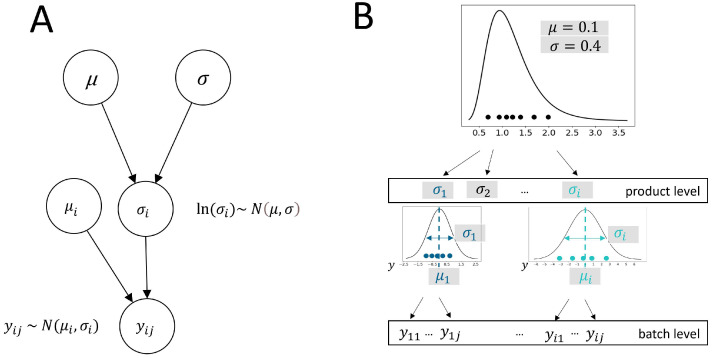
(**A**) Schematic representation of the random-variance model. The DAG shows the relationships of all parameters in the model and their distribution assumptions. Population parameters μ and σ parameterize a lognormal distribution, and product-specific variances ($\sigma_{i}^{2}$) are considered as random effects, where $ln\left(\sigma_{i}\right)∼N\left(\mu ,\sigma \right)$. The $\sigma_{i}$ parameters then determine the spread of normally distributed measures ($y_{ij}∼N\left(\mu_{i},\sigma_{i}\right)$). In a basic case of the model, each batch is considered to have only a single measurement (single-cycle). (**B**) Dataset generation process used in the simulation study. In the first step, product-specific standard deviations ($\sigma_{i}$) are drawn from a lognormal distribution with predefined parameters of μ=0.1 and σ=0.4. These sampled values determine the variances ($\sigma_{i}^{2}$) at the product level. In the second step, these variances are used to draw normally distributed data ($y_{ij}$) at the batch level. For each simulation dataset, both $\sigma_{i}$ and $y_{ij}$ are newly sampled.

The directed acyclic graph (DAG) representing the model framework in Figure 2A illustrates the dependence relationships of model parameters within the variance model framework. Each node in the DAG thereby represents a model parameter, and the directed edges (arrows) indicate the direction of dependency of parameters. The meta-analysis model involves the assumption that standard deviations of CQA measures of *i* products follow a lognormal distribution (see Figure 2B), i.e., $\sigma_{i}$ represents the considered random effects stemming from a population distribution with a mean of μ and a variance of $\sigma^{2}$. Thus, this random-effect variable in the model captures the random variations across product variances. This assumption of lognormality is not only plausible due to the non-negativity of variance but can also be inspected in a plausibility check using QQ plots. At the batch level of each product, the data collected from individual batches ($y_{ij}$) are modeled as samples that are assumed to follow a normal distribution, parameterized by the standard deviation ($\sigma_{i}$) and a fixed effect product mean ($\mu_{i}$). In the basic case of the model, as described in Figure 2, each batch is considered to have only a single measurement (single-cycle).

Within a biopharmaceutical platform process such as that described in Figure 1, upstream and downstream manufacturing of large-scale batches comprise a high volume of cell material. A purification step, such as affinity chromatography, is used to purify specific molecules from the harvested cell material. If the entire volume of harvested cell material cannot be processed in a single run, the purification is performed in cycles. In a cyclic purification process, the batch material is divided, and a certain amount is sequentially loaded onto the affinity chromatography column. For each cycle, analytical CQA results (e.g., ionic peaks in a mass spectrogram or aggregates) are obtained, providing measures across multiple cycles rather than a single-cycle measure.

**Figure 3:**
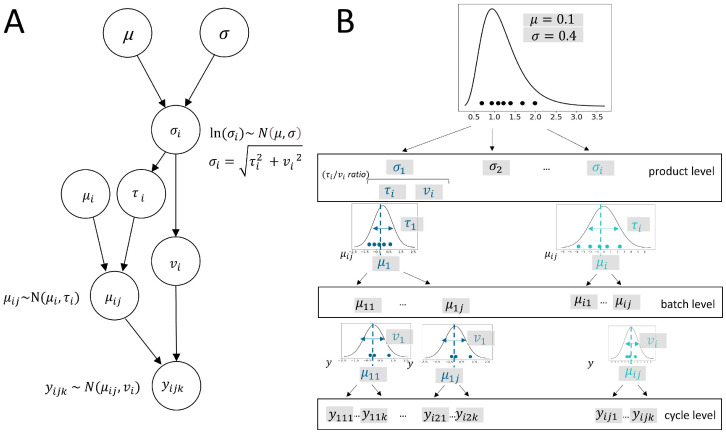
(**A**) Schematic representation of the random-variance model for multilevel data. The DAG shows the relationships of all parameters in the model and their distribution assumptions. As for the model for the reduced hierarchy structure (see Figure 2), μ and σ, as population parameters, parameterize the distribution over parameters $ln\left(\sigma_{i}\right)∼N\left(\mu ,\sigma \right)$. The multilevel model accounts for the additional batch-level, with $\mu_{i}$ and $\tau_{i}$ parameterizing the batch means ($\mu_{ij}∼N\left(\mu_{i},\tau_{i}\right)$). The product’s total standard deviation as random effects, thus, add up from the batch-to-batch variance ($\tau_{i}^{2}$) and residual variance across all batches ($v_{i}^{2}$) as separate variance components. These parameters determine the observed $y_{ijk}$ as a normally distributed random variable $y_{ijk}∼N(\mu_{ij},v_{i}$). (**B**) Data generation process used in the multi-cycle case of the simulation study. Similar to the sampling process for the single-cycle case, in the first step, product-specific standard deviations ($\sigma_{i}$) are drawn from a lognormal distribution with predefined parameters of μ=0.1 and σ=0.4. A predefined $\tau_{i}/v_{i}$ ratio of batch-to-batch and within-batch variance determines the variance components of total product variances ($\sigma_{i}^{2}$). On a batch level, for each batch *j*, a batch mean ($\mu_{ij}$) is sampled from a normal distribution with a product-specific $\mu_{i}$ and standard deviation $\tau_{i}$. Normally distributed data points ($y_{ijk}$) are then drawn as samples from a distribution with a product-specific, within-batch standard deviation of $v_{i}$. This within-batch variance component determines the residuals for multiple cycles within batches.

For the random-variance model, this implies that the hierarchical structure of products, batches, and cycles must be considered in terms of the resulting variance components, which are captured within the multi-cycle data. Therefore, the basic case of the random-variance model has to be extended for a multi-cycle data case, where each batch is considered to have several measurements (multiple cycles). Figure 3A provides a schematic representation of the random-variance model for multi-cycle data. This DAG expands on the single-cycle case shown in Figure 2A, including an additional level in terms of further model parameters and their dependencies within the model framework. Again, μ and σ, as population parameters, parameterize the distribution over parameters $ln\left(\sigma_{i}\right)∼N\left(\mu ,\sigma \right)$. The product’s total standard deviation is modeled as a random effect in the multi-cycle case. It comprises the batch-to-batch variance ($\tau_{i}^{2}$) and residual variance across all batches ($v_{i}^{2}$) as separate variance components estimated from cycle measurements. Model parameters $\mu_{i}$ and $\tau_{i}$ describe the batch means ($\mu_{ij}∼N\left(\mu_{i},\tau_{i}\right)$) sampled with product-specific average offsets ($\mu_{i}$) and batch variability ($\tau_{i}$). This way, the model appropriately includes the additional level of multiple *j* batches. The measured data points ($y_{ijk}$) finally result in a normally distributed random sample ($y_{ijk}∼N\left(\mu_{ij},v_{i}\right)$).

Within our model implementation in Stan probabilistic language (version 2.26.1) [21], we consider both described versions of the model such that product data from both cases can be integrated together in a meta-analysis.

### 2.2. Simulation Study

#### 2.2.1. Study Design Setting

We conducted a simulation study that effectively highlights the model and the robustness of its results. The chosen study design setting demonstrates the impact of dataset sample sizes on various aspects of the model. The results, detailing the observed effects for different numbers of products and batches, are presented in Section 3.1. The simulation study further comprises settings that demonstrate the model’s applicability to single-cycle and multi-cycle data. Results for multi-cycle simulation examples are presented in Section 3.2.

The simulation study, including all statistical analysis, was performed using R statistical software (version 4.2.2) [22].

#### 2.2.2. Data Generation

In a series of simulation studies, the model results were investigated for different sample sizes. Population parameters were set to fixed target values of μ=0.1 and σ=0.4. These predefined target values were chosen based on their fit to a real dataset, resulting in an variance distribution that represents the realistic magnitude of process variance observed within historical data for the given context of application. Datasets with different sample sizes were simulated, i.e., in each simulation study, a different sample size setting was used, with the number of products set to $n_{P}\in \left\{5,7,10,15,25,50\right\}$ and the number of batches set to $n_{b}\in \left\{3,5,7,10,15,25,50\right\}$. For each sample size setting, the simulation was repeated $n_{sim}=1000$ times. Each of the $n_{sim}$ simulation repetitions applied Bayesian inference to a newly generated dataset. The procedure to generate a simulation dataset is illustrated in Figure 2B. In each dataset, the total group variance per product is determined by $\sigma_{i}^{2}$, which is sampled from a lognormal distribution $ln\left(\sigma_{i}\right)∼N\left(\mu ,\sigma \right)$. Samples $y_{ij}$ are drawn for each product (*i*, where $y_{ij}∼N\left(\mu_{i},\sigma_{i}\right)$) with $\sigma_{i}^{2}$ representing random-effect variances, together with a product-specific fixed effect ($\mu_{i}$, set to zero for the simulation).

In this way, the random variances for individual products realize a different spread (i.e., varying broad sample distributions) of measures on the batch level (compare Figure 2B).

Importantly, the Bayesian hierarchical model is applicable for this statistical analysis, extending the location-scale modeling approach to multilevel data. To illustrate this, the simulation study is extended by an example case that incorporates a multilevel data structure (multi-cycle case). We demonstrate a simulation study setting with 10 products, each with 7 batches of 3 cycles per batch, which are representative of a real data case. The hierarchical data structure is characterized by its nested nature: cycles $k_{1\ldots n_{ij}}$ are nested within batches $j_{1\ldots n_{i}}$, which, in turn, are nested within products $i_{1...n}$. As in the previously described scenario, $n_{sim}=1000$ simulation runs were performed, and model predictions were assessed across all runs to confirm the validity of the model results for the multi-cycle data case. In each of the $n_{sim}$ repeated simulations, a Bayesian inference is applied to a newly generated dataset. To generate simulated data, in the initial step, total group variances are sampled from a lognormal distribution, denoted as $ln\left(\sigma_{i}\right)∼N\left(\mu ,\sigma \right)$. This variance comprises two components: the variance between batches ($\tau_{i}^{2}$) and the residual variance ($v_{i}^{2}$), which represents the variance within batches across all products. Within the model, these components are considered uncorrelated. The total variance, calculated as the squared sum of these two components, is expressed as $\sigma_{i}^{2}=\tau_{i}^{2}+v_{i}^{2}$. In the multi-cycle simulation showcased here, we varied the ratio of batch-to-batch to residual standard deviation ($\tau_{i}/v_{i}$). In this way, the influence of the ratios of the variance components on the model performance could be investigated. In the subsequent step, based on the random batch effects ($\tau_{i}$), batch means $\mu_{ij}∼N\left(\mu_{i},\tau_{i}\right)$ for individual batches within products are drawn. Alongside the residual variance ($v_{i}^{2}$), normally distributed samples ($y_{ijk}∼N(\mu_{ij},v_{i}$)) are drawn. This way, the generated dataset resembles real data measured in biopharmaceutical process development.

#### 2.2.3. Bayesian Parameter Inference and Predictive Distribution

In the Bayesian framework, each parameter in the random-variance model is characterized by a probability distribution, capturing the uncertainty in the parameter estimates. Initially, these distributions are represented as priors, reflecting our beliefs about the parameters before observing any data. Once data are observed, the prior is updated to form a posterior distribution that incorporates both the prior information and the new data (*Y*). This subsequent update of the priors is informed by the sample data, captured by the likelihood function (p(y|μ,σ)). As described in Section 2.1.2, the model includes population parameters μ and σ, which define the lognormal distribution of variances across products, and group parameters $\mu_{i}$ and $v_{i}^{2}$, which define the mean and variance for individual products, respectively. Population parameters μ and σ are the parameters of interest. For these parameters, we defined weakly informative prior distributions as standard normal distributions. This prior assumption was maintained for all simulation study settings. To evaluate the influence of the prior, an additional prior sensitivity analysis was conducted, showing no influence of the prior.

The statistical model described in Figure 3A is inferred via Markov Chain Monte Carlo (MCMC) sampling. The multivariate posterior distribution of the model parameters is thereby estimated by the no-U-turn sampler (NUTS) algorithm, as proposed by Hoffman and Gelman [23], implemented in Stan. This algorithm can efficiently sample from complex probability distributions, moving through the parameter space by automatically adapting the step size and number of steps according to the geometry of the target distribution. Bayesian parameter inference results in a multivariate posterior estimate. The posterior is multivariate because it accounts for the joint distribution of all model parameters, capturing the dependencies and correlations between them. MCMC sampling was conducted with two chains with a Markov chain length of 5000 sample draws (iterations), inclusive of 1000 warmup samples, which were discarded. To reduce autocorrelation between samples, a thinning factor of 2 was selected, meaning that every second sample was discarded from the chain. This resulted in an effective set of 4000 samples. Additionally, we set a target acceptance criterion of 0.95, a step size of 0.05, and a maximum tree depth of 15 to guarantee the efficiency and ergodicity of the Markov chain sampler. These settings were pre-determined to ensure they were sufficient for generating stable posterior distributions and were therefore set accordingly. To assess the model estimations for validity, Bayesian inference results were checked for MCMC convergence behavior. We used R package *shinystan* [24] to visually investigate the plots of sampling traces, as well as the autocorrelation and bivariate marginal densities of the posterior parameters. Furthermore, we checked for a Gelman–Rubin statistic ($\hat{R}$) close to 1 as an indication of convergence and mixing of the chains. $\hat{R}$ measures the convergence of multiple Markov chains by comparing the variance within each chain to the variance between chains, with values close to 1 indicating that the chains have converged to the same stationary distribution. The effective sample size ($n_{eff}$) serves as an additional diagnostic tool for sampling, measuring the efficiency of samples in providing information for parameter estimation by considering the correlation between samples. It represents the number of independent samples that would yield the same level of precision as the correlated samples. To ensure sufficient sampling across all simulation settings, we verified that $n_{eff}$ was close to 4000 as the total number of posterior samples used for estimation.

To assess the uncertainty of the parameter mean estimates for $\hat{\mu }$ and $\hat{\sigma }$ across simulation studies, we determined 95% intervals from 10,000 bootstrapped MCMC posteriors (of size $n_{sim}$).

The posterior predictive distribution is used to predict future product variance based on the observed data, considering the uncertainty about the model parameters. Based on the marginal posterior distribution (p(θ|Y)), the posterior predictive distribution for a future total variance ($S^{2}$) is expressed as

(1) $$p\left(S|Y\right)=\int_{\theta }p\left(S|\theta \right)p\left(\theta |Y\right)d\theta ,$$

where the parameter vector is θ=(μ,σ), the standard deviation estimate is *S*, and data are represented by *Y*. This posterior predictive distribution is the resulting predictive model of interest, derived from the meta-analysis. It provides quantitative estimates of process variances and their uncertainty, which can be included in further statistical calculations, e.g., for determining process ranges.

#### 2.2.4. Performance Criteria

The empirical standard error (SE) across parameters mean parameter estimates of all simulation studies was calculated as $\sqrt{Var(\hat{\mu })}$ and $\sqrt{Var(\hat{\sigma })}$. The coverage of credible intervals (CIs) and coverage of prediction intervals (PIs) were computed based on the simulation repetitions. Non-parametric bootstrapping with replacement was performed to estimate the uncertainty of the coverage estimation. The bootstrap interval was determined by taking the 2.5% and 97.5% percentiles from this distribution, providing an estimate of the interval within which the true parameter value lies with 95% probability. To evaluate the sensitivity of the results to the chosen prior distributions and, thereby, assess the robustness of the model predictions, a prior sensitivity analysis was conducted. The prior distributions for the population parameters (μ and σ) were systematically varied. Initially, the standard normal distribution with a mean of 0 and a standard deviation of 1 was used as the baseline. Subsequently, the analysis was extended to include wider distributions with standard deviations of 2, 3, 4, and 5. In this way, we systematically explored the impact of less informative priors on the Bayesian inference results. The prior sensitivity analysis can confirm that the model results are well-supported, regardless of the specific prior distributions. The analysis shows that the chosen symmetric distribution represents a weakly informative prior assumption that serves as a starting point, ensuring that the data primarily drive the inference.

## 3. Results

The analysis results of the simulation study are used to evaluate the performance of the model. The choice of simulation settings, defined by the number of products ($n_{P}$) and number of batches ($n_{b}$), allows for investigation into the effect of sample size on the model outcome and its robustness. A multilevel data example was provided to extend the simulation study to a case study with multiple cycles per batch. The analysis of different $\tau_{i}$ and $v_{i}$ ratios allowed for the investigation of the sensitivity of the parameter accuracy and model coverage for unequal variance composition.

### 3.1. Investigation of Sample Size Effect

#### 3.1.1. Parameter Inference

**Figure 4:**
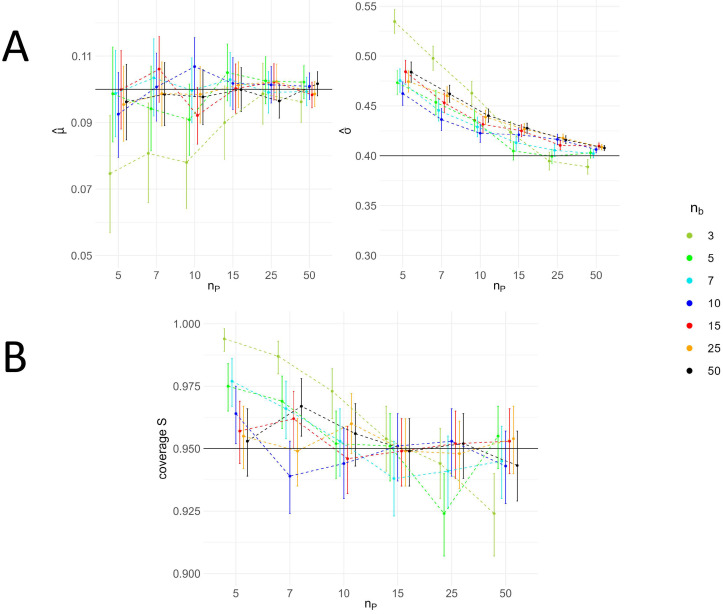
Simulation study results on single-cycle data for different sample sizes. (**A**) Mean population parameter estimates presented together with 95% bootstrap intervals. The effect of sample size in the simulated datasets, i.e., the number of products ($n_{P}$) and number of batches ($n_{b}$) based on the inferred population parameters ($\hat{\mu }$ and $\hat{\sigma }$) is reflected in the calculated mean estimates across simulation samples. As the sample size increases, the mean parameter estimates approach the true values of μ and σ. The data points along the x-axis are jittered to enhance their distinguishability. (**B**) Coverage of 95% prediction intervals (PIs) presented together with 95% bootstrap intervals. The coverage, demonstrating the model’s prediction validity on $s_{P}$, approximates 95% for most of the simulation study scenarios. However, smaller sample sizes tend to yield broader PIs, indicating increased uncertainty in the model’s predictions. Undercoverage occurs only in a few cases and is not considered a systematic effect. The 95% bootstrap intervals depicted in the figure represent the Monte Carlo error associated with the simulations. The data points along the x-axis are jittered to enhance their distinguishability.

Posterior mean estimates of $\hat{\mu }$ and $\hat{\sigma }$ averaged across all simulation studies are plotted as a function of number of products ($n_{P}$) and compared for different numbers of batches ($n_{b}$) (see Figure 4A). With a smaller sample size, there is less information available to accurately estimate the parameters. In a Bayesian framework, this increased uncertainty is reflected in the posterior distribution of the parameters, leading to more spread-out probability distributions. Consequently, large values for μ and σ have a non-negligible probability, resulting in higher expected values for $\hat{\mu }$ and $\hat{\sigma }$. This expected overestimation bias is evident in our simulation results when $n_{P}$ and $n_{b}$ are small. For $n_{b}>3$, the estimate of the lognormal expected value ($\hat{\mu }$) closely approximates μ. However, the estimated standard deviation ($\hat{\sigma }$) tends to overestimate the true σ.

**Figure A1:**
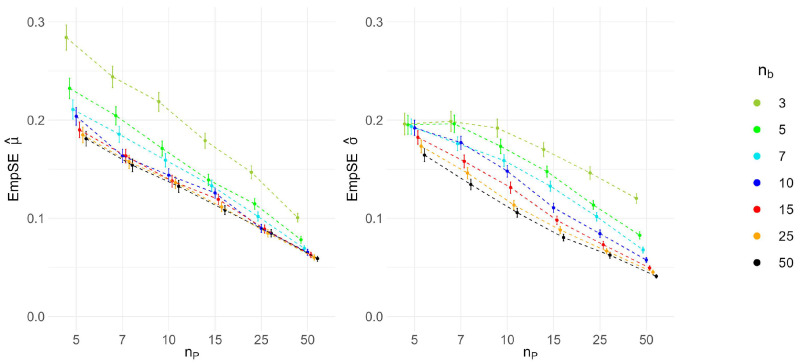
Simulation study results on single-cycle data for different sample sizes. The empirical standard error (EmpSE) of $\hat{\mu }$ and $\hat{\sigma }$ is calculated as the standard deviation of the mean estimates obtained from multiple simulated datasets. It shows a decrease with an increase in sample size. The 95% bootstrap intervals depicted in the figure represents the Monte Carlo error associated with the simulations. The data points along the *x*-axis are jittered to enhance their distinguishability.

These results provide an important reference for selecting a meta-analytic dataset, indicating the sample size required to achieve unbiased inference results. As $n_{P}$ increases, the uncertainty decreases, leading to smaller bootstrapped intervals. The relative biases ($E[\hat{\mu }]-\mu$ and $E[\hat{\sigma }]-\sigma$) of the population parameters (μ and σ) decrease with an increasing number of products and batches. Simulation settings with $n_{b}\geq 5$ can already cover the true mean μ within the uncertainty intervals of the estimated parameter. The tendency for overestimation of the true standard deviation (σ) observed in the simulation study results is ultimately reflected in the predictive distribution (as described in Section 2.2.3). Empirical standard errors (EmpSEs) of μ and σ increase with lower sample sizes (see Figure A1), reflecting the uncertainty in parameter estimations across simulation studies.

#### 3.1.2. Model Coverage

The coverage probabilities of CIs for μ and σ, as well as the coverage probability of the PIs for standard deviations (*S*), serve as key performance metrics indicating the model’s reliability and accuracy. The coverage quantifies how well the 95% uncertainty intervals capture the true values, and it is expected to be close to the targeted value of 95%. In all simulation scenarios, the coverage of the CI for both parameters of interest ranged between 94.2% and 98.5%. Notably, lower sample sizes were associated with higher coverage for both parameters.

Additionally, the coverage probability of PIs across all simulation scenarios remained within an acceptable range close to the targeted value (92.4–99.4%), as illustrated in Figure 4B. This indicates that the predictive distribution is an appropriate model for deriving uncertainty ranges, such as the 95% PI, for future observations of standard deviations (*S*). Higher coverage observed with $n_{P}$ and $n_{b}\leq 7$ suggests model bias, reflecting increased uncertainty due to small sample sizes. Bootstrap intervals account for simulation uncertainty and include the targeted value of 95% in most cases. However, in scenarios with a small $n_{b}$, the bootstrapped intervals do not include the targeted 95%. The resulting coverage of the PI indicates the method’s effectiveness in predicting standard deviations (*S*) for future products.

Furthermore, we conducted a prior sensitivity analysis, which confirmed that the inference of the model parameters is not significantly influenced by the choice of prior distribution.

### 3.2. Model Inference on Multilevel Data

In this simulation study, we also examined the model’s performance in a multilevel example case. The simulation settings were selected based on typical averages for the number of products, batches, and cycles per batch, as observed in previous biopharmaceutical process development. Consistent with the results from the single-cycle simulation settings, the resulting coverage metrics offer a comprehensive understanding of the model’s performance. Additionally, this study elucidates how varying ratios of the variance components (i.e., the $\tau_{i}/v_{i}$ ratio) affect parameter inference and model prediction.

**Figure 5:**
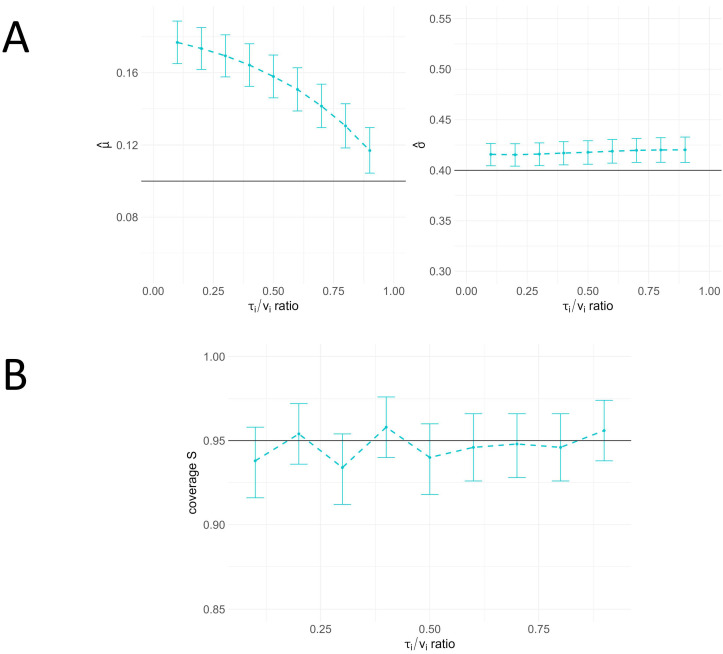
Simulation study results on multi-cycle data for different $\tau_{i}/v_{i}$ ratios. (**A**) Mean values for parameters μ and σ in a simulation involving three products, ten batches, and three cycles per batch presented with 95% bootstrap intervals. The estimates are compared across different $\tau_{i}/v_{i}$ ratios. While variations in the $\tau_{i}/v_{i}$ ratio affect the mean estimates for σ, a smaller within-to-batch variance ($\tau_{i}^{2}$, resulting in a smaller ratio) leads to higher mean estimates for μ. (**B**) Coverage of 95% prediction intervals (PIs) for the standard deviation (*S*) in the same simulation setup remains stable around the expected value of 95% across different $\tau_{i}/v_{i}$ ratios.

**Figure A2:**
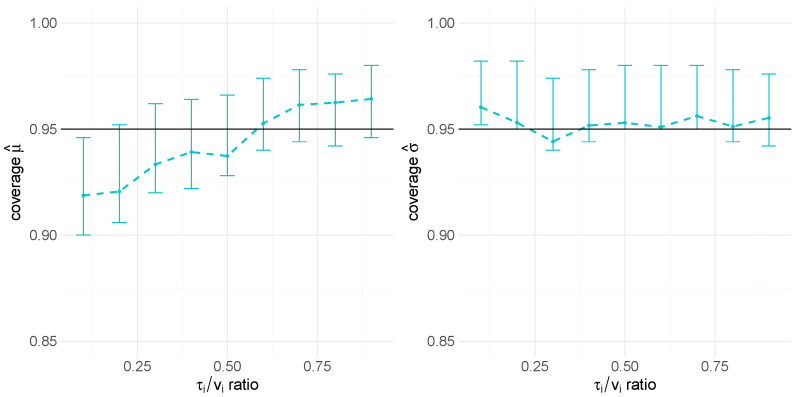
Simulation study results on multi-cycle data for different $\tau_{i}/v_{i}$ ratios. Coverage of 95% credible intervals (CIs) is presented for parameters μ and σ, together with 95% bootstrap intervals. The CI coverage for σ remains unaffected by variations in the $\tau_{i}/v_{i}$ ratio. However, a smaller within-to-batch variance ($\tau_{i}^{2}$, resulting in a smaller ratio) leads to slightly underestimated CI bands for μ.

The mean parameter estimates for μ and σ, along with the coverage of the CI, are shown in Figure 5 and Figure A2. Similar to the inference results on single-cycle data, the mean posterior of $\hat{\sigma }$ is higher than the true value and remains constant for different $\tau_{i}/v_{i}$ ratios (see Figure 4A). The mean posterior estimate of $\hat{\mu }$ is consistently higher than the true values across all cases in multilevel model inference. This bias is in contrast to the results on single-cycle data (see Figure 4) and appears to increase with a higher between-batch variance component ($\tau_{i}$). As illustrated in Figure A2, a higher between-batch variance ($\tau_{i}$) relative to within-batch variance ($v_{i}$) increases the coverage of $\hat{\mu }$, while the coverage for $\hat{\sigma }$ remains constant. With a higher $\tau_{i}/v_{i}$ ratio, the coverage of $\hat{\mu }$ increases, and the bootstrapped uncertainty intervals around the coverage include the target 95% value in nearly all cases. The multilevel simulation example demonstrates that the coverage of the PI is close to the expected 95% across all simulation runs for different $\tau_{i}/v_{i}$ ratios (see Figure 5B).

As previously mentioned, the predictive distribution ($p(S|\hat{\mu },\hat{\sigma })$) is the targeted model for estimating cross-product variance. The coverage of the PI is therefore the most important metric to demonstrate the model’s ability to provide accurate estimates of future process variance. Therefore, the results suggest that the Bayesian hierarchical model can serve as a valid tool to be used in statistical analysis for meta-analysis of historical biopharmaceutical process data.

## 4. Discussion

In this study, we introduced a novel Bayesian hierarchical model designed to estimate process variability in biopharmaceutical data. This model framework facilitates the analysis of variances within a meta-analysis, assuming a global distribution at the population level. We developed a multilevel model that employs Bayesian statistical inference to derive population parameters, describing platform process variability across historical products. The Bayesian framework is particularly suitable for our objective, as it is highly effective in characterizing variability from limited data [25].

Our approach expands traditional meta-analysis approaches used for between-study variance estimation in clinical trials. The modeling framework shares similarities with clinical trial meta-analysis, where heterogeneity (τ), as a scale parameter, relates to between-study differences [26], which can be estimated and, thus, inform future predicted variability. This can be seen as analogous to the product-to-product variability within our CMC biopharmaceutical application case. However, our proposed hierarchical model provides a predictive distribution of variances, from which prediction intervals can be directly derived. Unlike conventional methods that often assume the homogeneity of effect sizes (i.e., the magnitude of the difference between treatment and control) and, thus, comparable variability between groups (for a summary and comparison of estimators, see Veroniki et al. [12]), our random-effect model accounts for heteroscedasticity and dependencies between measures of the same batches. This adds an additional hierarchical level that accounts for within-batch variance. The multilevel structure could be modeled within frequentist hierarchical mixed-effect models [27]. These approaches are particularly useful when dealing with nested data but do not inherently provide a predictive distribution in the way Bayesian models such as our random-effect model do. Furthermore, for our intended statistical model application and purpose, a significant advantage of the Bayesian approach is seen in its ability to integrate the predicted distribution of process variance directly as prior information. Bayesian non-parametric methods, such as Dirichlet process mixtures or Gaussian process regression [28,29], can account for variability across groups. While they share similarities with random-effect models, their advantage lies in their ability to grow in complexity by adapting the flexible number of parameters. This feature is particularly useful when the number of groups is unknown, although it is not the case in our application. Similar to our newly proposed approach that provides a hierarchical framework, other Bayesian random-effect models, such as network meta-analysis [30], also account for heterogeneity between groups. These model may be applicable to the hierarchical data in our application scenario. However, these models do not share the same objective as ours and are more readily used for determining effect sizes rather than providing predictive variance distribution for future products.

In comparison to these random-effect models that focus on between-group variance, our approach differs in that it includes the total variances of products as random effects. The model enables prediction of cross-product variance, making it applicable to new drug products. This represents a real advantage of our random-effect model of variance over fixed-effect models, which only provide estimates for individual products.

Our newly developed method shares foundational similarities with location-scale modeling [17,18,19,20,31].

Importantly, our approach extends existing models to handle hierarchical data structures. Our simulation study demonstrates that the model can manage both single-cycle and multi-cycle data—and, crucially for real-case meta-analysis, a mixture of both. This introduces a novel meta-analysis approach, broadening the applicability of location-scale models to multilevel meta-analysis within the biopharmaceutical context. This new approach is highly relevant because current CMC statistics face the challenge of scarce data, which introduces uncertainty to variability estimates of CQAs measured across unit operations in both upstream and downstream processes. An important aspect frequently discussed in the context of clinical statistics is the number of samples (in our case, the number of products and number of batches). This is particularly crucial when dealing with scarce data, as in our application. In such cases, a point estimate for the variance is affected by high uncertainty. To account for this uncertainty, several methods have been proposed [32,33,34], which provide confidence intervals and facilitate variance interpretation [35]. Unlike our approach, these methods do not consider the variance itself as a random effect.

The simulation study results for the multilevel data revealed a bias in the mean parameter estimate and the coverage of the CI for high between-batch variance (i.e., low $\tau_{i}/v_{i}$ ratio). Although the bootstrap intervals for the coverage CI include the target 95% value, the resulting posterior mean of the mean parameter of the lognormal distribution is increased. However, this overestimation bias is not reflected within the predictive distribution. Therefore, the results still support the validity of our random-variance model for multilevel data.

In the simulation study, we generated data based on the model, assuming $\sigma_{i}$ as the total variance of products follow a lognormal distribution. As an alternative, the variance components at the level of batches ($\tau_{i}^{2}$) and cycles ($v_{i}^{2}$) could also be assumed to follow this non-negative distribution. However, we are not interested in their distributions because only the distribution of the total variance is of interest for biopharmaceutical applications. Thus, we do not demand them to be random but fixed effects, eliminating the need for additional distributional assumptions for both parameters, as well as their correlation. This means that for our simulation, as well as real-world data, no test for correlation is necessary.

To assess the robustness of the model inference, we can compare the quantiles of the mean posterior estimates of $\sigma_{i}$ from the hierarchical model to the theoretical quantiles of a lognormal distribution using a QQ plot. A close alignment of the points along the reference line indicates that the assumption of $\sigma_{i}$ being lognormally distributed is well-supported by the simulation study results. In real data analysis, it is crucial to visually check the plausibility of the population distribution assumption to verify whether the products are suitable for evaluation together in the meta-analysis. To check the plausibility of the assumption that $\sigma_{i}$ is lognormally distributed, standard deviations calculated for individual products can be evaluated using a QQ plot. This visual confirmation is important in real data analysis before evaluating model inference results and can support the model’s underlying assumption. Within a real-world data analysis, it would be beneficial to use goodness-of-fit tests, such as the Kolmogorov–Smirnov test or the Anderson–Darling test, to further validate the distributional assumption.

Another assumption made by the model is that variance components are uncorrelated. This assumption is plausible within the context of CMC in biopharmaceutical processes. Batch variability in biopharmaceutical manufacturing can arise from several sources, particularly differences in seed trains. When comparing different seed trains, variability can stem from factors such as raw materials, medium composition, culture conditions, and process parameter settings. Although these factors are maintained within strict and controlled ranges, small differences can lead to variations in CQA measurements. Within-batch variance, which can be assessed by measuring multiple cycles per batch, has different sources. It encompasses the measurement of various parts of the overall material and pure analytical variance, such as equipment performance and operator techniques.

As mentioned before, the proposed model has several practical implications for CMC biopharmaceutical drug development and manufacturing. Upstream and downstream processing of biopharmaceuticals involves multiple steps, including cascaded cell cultivation, harvest, and purification. During these unit operations, CQAs define the required product quality. Variance estimation for analytical measurements of a CQA at a corresponding operating unit is typically performed and used, for example, to derive specification limits. Our simulation study’s prediction interval coverage results demonstrate that the model provides valid results for both single-cycle and multi-cycle data, as well as for different ratios of variance components. By incorporating historical data, the Bayesian model can be applied, for instance, within an MAP prior approach, where the predicted distribution of historical variances could be included as prior knowledge for inference on new data. Integrating the predictive distribution in future statistical analyses would appropriately account for differences between products while borrowing information to infer variance parameters for individual products. This way, the approach allows for more accurate variance estimates of CQAs in platform processes, which is crucial for quality control and process optimization. Establishing a comprehensive methodological workflow that applies the proposed meta-analysis framework can provide significant support for future product development. Leveraging existing platform process data minimizes the need for extensive new experiments, saving both time and resources and thereby promoting sustainability. More accurate variance estimates enable better-informed decisions during the early phases of process development, potentially accelerating the overall development timeline. Variance estimation can also be a useful tool to evaluate models, such as for the design of experiments, providing references for expected variances and ensuring process modeling quality.

Our simulation studies covered a range of sample sizes. However, real-world data may present additional complexities. In a real-data meta-analysis, products should be critically assessed with regard to their similarity (e.g., molecule types) beforehand. This pre-assessment of a dataset prevents the distortion of variance estimation due to outliers that do not reflect the true variance of the platform process.

The model assumes that the data follow a lognormal distribution, which may not always hold true in practice. Future research could explore the applicability of other distributions.

In future studies, various methodological approaches to applying the model could be explored. The model can directly predict process variance across products based on meta-analytic data. The simulation study introduced and explained the model as a hierarchical random-variance model, which is highly valuable for CMC in biopharmaceutical applications. Additionally, the inferred distribution could be employed as an empirical MAP prior, as mentioned in the Section 1. This allows for a detailed comparison of different Bayesian borrowing approaches, which are well-known in clinical statistics [36]. For biopharmaceutical applications, the new model approach presented here can support predictions for process variance in individual unit operations across the production chain of a drug product. A further direction for future work would be the extension of our hierarchical random-variance model to time-series data. Location-scale models of hierarchical time series data would bring additional benefits for upstream process development and manufacturing.

The work presented in this study is an important step in these directions. By leveraging our historical platform knowledge, we can highlight the value of our data in advancing CMC’s future biopharmaceutical drug development and ensuring quality for patients.

## 5. Conclusions

The Bayesian hierarchical model extends location-scale models to hierarchical data relevant for biopharmaceutical development. It was implemented and validated through a simulation study, demonstrating its effectiveness in leveraging historical data to improve the estimation of process variance. The simulation study results can confirm the model’s accuracy and reliability across various sample sizes. Based on these results, the proposed hierarchical model shows promise and utility for enhancing statistical analysis in CMC drug development. The model provides prediction of expected platform variability and can be included in calculations for new process variances early on. Our model represents an important support for statistical analysis of scarce data. Thus, it is particularly valuable in addressing current challenging situations where few data are available from large-scale batches. This underscores the model’s applicability for meta-analysis of real-world upstream and downstream CQA measures in biopharmaceutical applications.

## Data Availability

The raw data supporting the conclusions of this article will be made available by the authors on request.
